# Nano-enhanced bioremediation of soil co-contaminated with petroleum hydrocarbons and cadmium using *Helianthus annuus* L. plants

**DOI:** 10.1186/s12870-026-09746-6

**Published:** 2026-08-13

**Authors:** Visha Habib, Muhammad Imran Khan, Qammar Farooq, Muhammad Hayder Ali, Hafiz Naeem Asghar, Muhammad Naveed, Majed Alotaibi, Nawab Ali, Mahmoud F. Seleiman

**Affiliations:** 1 Institute of Soil and Environmental Sciences, University of Agriculture, Faisalabad, 38040 Pakistan; 2 Department of Forestry, College of Landscape Architecture, Sichuan Agricultural University, Chengdu, China; 3 Plant Production Department, College of Food and Agriculture Sciences, King Saud University, P.O. Box 2460, Riyadh, 11451 Saudi Arabia; 4 Department of Biosystems and Agricultural Engineering (BAE), College of Agriculture and Natural Resources, Michigan State University, East Lansing, MI USA

**Keywords:** Nanotechnology, Phytoremediation, Heavy metals, Organic pollutants, Microbes, Oilseed crops

## Abstract

**Background:**

Simultaneously removing petroleum hydrocarbons (PHCs) and heavy metals from soil is a challenging task. This study aimed to evaluate the efficacy of green-synthesized nano zero-valent iron (nZVI) particles and a microbial consortium in combination with sunflower (*Helianthus annuus* L.) plants for nano-enhanced bioremediation of soil co-contaminated with PHCs and cadmium (Cd). A pot trial was conducted by growing sunflower plants on soil co-contaminated with 6000 mg kg^-1^ and 30 mg kg^-1^ of PHCs and Cd, respectively, and nZVI particles and a microbial consortium were added to the soil for plant growth enhancement and pollutants removal.

**Results:**

The results revealed that the co-contaminated soil caused significant phytotoxicity to sunflower plants. However, the co-application of a microbial consortium and nZVI particles considerably alleviated the phytotoxic impacts of Cd and PHCs on sunflower plants, demonstrating up to 72.7% and 83.5% improvements in plant physiology and growth in contaminated soil, respectively compared to the respective unamended control. Furthermore, the combined application of a microbial consortium and nZVI particles caused 59.4% and 58.6% reduction in bioavailable Cd, and 82.7% and 74.7% decrease in PHCs in planted and unplanted treatments, respectively, with respect to their initially applied concentrations.

**Conclusion:**

The findings from this study indicate that the integrated application of nZVI particles and a microbial consortium was very effective in improving plant growth, mitigating Cd-accumulation in plants, confirming Cd-immobilization, and removing PHCs from soil, highlighting the potential of nano-enhanced bioremediation as a sustainable solution for the treatment and management of soil co-contaminated with inorganic and organic pollutants.

**Supplementary Information:**

The online version contains supplementary material available at https://doi.org/10.1186/s12870-026-09746-6.

## Introduction

Environmental pollution by organic and inorganic contaminants is a worldwide concern that threatens the human health and well-being of all living organisms [1, 2]. Petroleum hydrocarbons (PHCs) are compounds composed of carbon and hydrogen atoms arranged in various molecular structures, typically present in crude oil, fuels, and many other refined petroleum products [3]. Oil and petroleum products are the most employed energy sources in all industries; yet their extraction, transportation, and usage are all related to environmental pollution. The major problem with oil-related pollution is that hydrocarbons have low biodegradability, threatening biodiversity and ecosystems [4]. Among inorganic pollutants, entry and accumulation of heavy metals in the biosystem is the major environmental concern that needs special attention [5]. For instance, cadmium (Cd), a non-essential trace element and a widely distributed metal, is often released in the environment by human activity, posing a significant impact on all living biota, including humans [6]. The main sources of Cd are fertilizers and pesticides, aside from other natural sources that include mining and atmospheric deposition from combustion emissions [7]. In addition to being a toxic metal, Cd has several harmful effects and low nutritional value for plants at various growth and development stages [6]. Also, once it enters the human body via the food chain, it can cause diseases like cancer and damage to the nervous system [8]. In addition, the mixed soil pollution, by the presence of both organic (e.g. PHCs) and inorganic (e.g. Cd) pollutants, often exacerbates the biotoxicity to the environment, and therefore, the treatment of such soil also remains more challenging [9].

In co-contaminated soils, the interaction between PHCs and Cd is governed by complex physicochemical and biological mechanisms that significantly influence their environmental fate, mobility, and toxicity [10]. PHCs, being hydrophobic organic compounds, can alter soil physicochemical properties by increasing organic carbon content and modifying soil surface characteristics, which in turn affects the adsorption and speciation of heavy metals such as Cd [11]. This interaction may enhance Cd immobilization through complexation with organic matter or increase its mobility depending on soil conditions such as pH, redox potential, and organic matter composition [12]. Conversely, Cd can exert toxic effects on hydrocarbon-degrading microbial communities by disrupting enzymatic activity and cellular functions, thereby reducing PHC biodegradation efficiency [13]. In addition, PHCs may also influence Cd bioavailability by affecting microbial-driven processes such as biosurfactant production and extracellular polymeric substance (EPS) formation, which can bind or mobilize metal ions [14]. Therefore, the coexistence of Cd and PHCs often leads to altered contaminant bioavailability, increased oxidative stress in biota, and reduced remediation efficiency compared to single-contaminant systems [15]. It has been stated that a site contaminated with multiple pollutants is more difficult to remediate than one contaminated with a single pollutant [16]. Among available options, the combined application of microorganisms with nanotechnology in plant-mediated remediation may have a promising potential for the treatment of soils co-contaminated with Cd and PHCs [17].

Different mechanisms are adopted by microorganisms for heavy metal tolerance, such as regulatory transport, compartmentalization, and siderophore [18–21]. Some of the important features, such as rapid growth and different functional groups on the surface of bacteria, such as *Bacillus subtilis*,* Escherichia coli*, and *Stenotrophomonas* sp., are known to enhance the ability of bacteria for precipitation of heavy metals in soil without affecting the soil fertility [22–25]. Likewise, microbes can also degrade PHCs, particularly by producing biosurfactants and hydrocarbon-degrading enzymes, including oxygenases and peroxidases that enhance the bioavailability and breakdown of pollutants [26]. Plant growth-promoting rhizobacteria further aid plants by nitrogen fixation, phosphate solubilization, and production of phytohormones like indole acetic acid (IAA), improving plant resilience in contaminated soils [27]. The beneficial relationship between plants and microbes is an important aspect for their adaptation to contaminated environments [28]. The synergistic interaction between plants and microbes can be further utilized in the microbe-assisted phytoremediation [28], and these synergistic effects in the bioremediation process can be further improved by the application of nanotechnology (such as nanoparticles) [29].

In recent decades, attention towards nano-bioremediation has increased due to its eco-friendly nature and cost-effectiveness [30]. The nano zero-valent iron (nZVI) (1–100 nm) particles with a smaller size and large specific surface area help in the remediation of contaminated soil by degrading contaminants through sharing their electrons [31]. The nZVI particles have been reported to transform the organic contaminants effectively in recent studies [31–33]. Previously, various plants have been used to produce nZVI particles; however, the presence of phytochemicals in mulberry leaf extract (e.g., polyphenols and flavonoids) facilitates the reduction of iron ions to Fe⁰ and acts as capping agents to stabilize the nanoparticles and prevent aggregation [32]. The optimization of iron oxide nanoparticles by different physicochemical surface properties, porous media diffusion, adsorption, and minimizing aggregation are some of the characteristics of nZVI particles that can further enhance the remediation process [34]. The nZVI particles play an important role in several adsorption processes, such as aggregation, ion exchange, precipitation, hydroxylation, and redox reactions, by interacting with different physical and chemical characteristics of heavy metal ions [35]. Despite their positive role in enhancing contaminant removal, the inclusion of nZVI particles can exert antimicrobial effects at higher concentrations due to oxidative stress and membrane damage. Therefore, it is crucial to use an optimized concentration of nZVI which is essential for developing a synergistic and mutually beneficial interaction with microbial communities. Here, nZVI facilitates contaminant transformation while microbial activity further enhances contaminant removal [36, 37].

Sunflower (*Helianthus annuus* L.) plants support human and animal diets, particularly due to their nutritional oils and seeds, and also offer significant environmental benefits, such as phytoremediation of pollutants in soil, due to their easy maintenance and fast development cycle [38]. Moreover, sunflower plants, due to their root exudates and enhanced oxygenation, boost microbial activities, improve soil conditions, and eventually accelerate pollutant breakdown and remediation [38]. The literature review elucidates that studies have reported (i) Cd uptake and tolerance in sunflower [38, 39], (ii) microbial degradation of hydrocarbons [28]; however, no studies have reported the simultaneous remediation of mixed pollutants (both organic and inorganic) using sunflower plants. Hence, it would be interesting to explore the potential of sunflower plants to remediate Cd and PHCs from soil through nano-enhanced bioremediation [40]. Furthermore, the synergistic interactions of nZVI and microbes can be employed to enhance plant physiology and overall growth by improving water and nutrient uptake, regulating physiological and biochemical processes, and subsequently strengthening the remediation capabilities of the sunflower plants [41]. However, till now, no study has reported the integrated application of nZVI particles, a microbial consortium, and plants for the remediation of soil co-contaminated with Cd and PHCs. Therefore, to address this gap, this study aimed to evaluate the individual and combined effects of nZVI particles and a microbial consortium on pollutant remediation and growth promotion of sunflower plants in soil co-contaminated with PHCs and Cd.

## Methods

### Green synthesis of nano zero-valent iron particles

The nZVI particles were synthesized in accordance with the method mentioned by Poguberović et al. [42]. The mulberry leaves were employed due to their high content of antioxidant phytochemicals, as well as their non-toxic, environmentally friendly, and cost-effective properties. They were obtained from the botanical garden of the University of Agriculture, Faisalabad. The leaves were dried in an oven at 50 °C for 48 h. The dried material was crushed by a kitchen chopper and sieved with a 2 mm mesh size. A 300-mL Erlenmeyer flask was used for the extraction process, by adding 3.7 g of leaf powder to 100 mL of distilled water and transferring it on a shaker at 80 °C for 60 min. Afterwards, a vacuum filtration pump was used to obtain the filtrate of the leaf extract solution, which was further used in the formation of nZVI particles. Following this, a 0.1 M FeCl_3_ solution was prepared and mixed in a 3:1 ratio with the extract. After the incorporation of the FeCl_3_ solution, the color changed to black, which indicated the presence of nZVI particles. To preserve the reactivity and zero-valent iron core, the freshly synthesized nZVI particles were immediately applied to soil (within approximately 10–15 min of preparation), to minimize their exposure to atmospheric oxygen and prevent premature oxidation. This rapid application ensured maximum availability of reactive surface sites and enhanced remediation efficiency in the soil system.

### Characterization of nZVI particles

A scanning electron microscope (SEM) (Emcraft CubeSeries, South Korea) was used to determine the surface morphology of nZVI particles. Clear and detailed images were acquired under operating conditions of 8 kV voltage and 1.203 mA current [43] (Figure S1a). The Fourier transform infrared spectroscopy (FTIR) was performed in the spectral range of 4000 –650 cm^− 1^ using a Cary 630 instrument (Agilent Technologies, USA) to identify the functional groups of the synthesized nanoparticles, as shown in Figure S1b [44]. An average of 32 scans per sample was obtained at a resolution of 4 cm^− 1^ to achieve an improved signal-to-noise ratio [45].

The X-ray diffraction (XRD) spectra were obtained by using a D8 Advance Bruker X-ray diffraction spectrometer (Billerica, MA, USA) at a scanning rate of 0.05 min^-1^, equipped with a scintillation counter using Cu Kα radiation (k = 1.5405 Å) and were scanned at 2θ range from 10–70° [46–48] (Figure S1c). Furthermore, the zeta sizer (Litesizer 500 Anon Paar Company, Austria) was used to assess the particle size distribution of nZVI particles by using the principle of Agarwal et al. [49]. With slight modifications, the nZVI particles were mixed with deionized water in a test tube with a concentration of 1 mg mL^-1^ and sonicated at 35 kHz for thirty minutes to achieve a homogenous suspension. After cooling to room temperature, the suspension was passed through a syringe filter (0.45 μm), to remove undissolved particles, and the filtrate was transferred into cuvettes. A single-frequency laser was directed to the sample to obtain the particle size (Figure S1d).

### Preparation of microbial consortium

The three pre-isolated identified microbial strains having accession numbers ON7114534 (*Acromobacter xylosoxidans* strain MH-13), ON7114535 (*Stenotrophomonas* sp. strain MH-18), and ON714537 (*Stenotrophomonas rhizophila* strain MH-24) were obtained from the Soil and Environmental Microbiology Laboratory (SEML), Institute of Soil and Environmental Sciences (ISES), University of Agriculture, Faisalabad (UAF). As per the protocol mentioned by Afzal et al. [50] and Ali et al. [51], the strains were cultured in equal proportions (1:1:1) in autoclaved tryptic soy broth (TSB) media under sterilized conditions and placed on a shaker at 28 ± 2 °C and 180 rpm for 72 h. The microbial growth was monitored by measuring optical density at 600 nm using a spectrophotometer (VS-1100, Biotechnology Medical Services, China), and the optical density was adjusted to 0.5 to standardize the bacterial inoculum (10^8^-10^9^ CFU mL^-1^). The survival, proliferation and colonization efficiency of the inoculated microbial strains in soil were not directly quantified in this study. However, uniform inoculation and controlled experimental conditions were maintained to support microbial establishment and activity in the soil matrix. Moreover, previous studies have reported that *Achromobacter* and *Stenotrophomonas* are well adapted to contaminated environments and can effectively colonize the rhizosphere [51, 52].

### Soil sample collection and contamination

The soil was collected from the research area of the ISES, UAF. The soil was dried under ambient air and sieved through a mesh size of 2 mm. Subsequently, the soil was artificially contaminated with cadmium chloride (30 mg kg^-1^) and diesel (6000 mg kg^-1^), which were used as sources of cadmium (Cd) and petroleum hydrocarbons (PHCs) in soil, respectively. The cadmium chloride and diesel were obtained from SEML of ISES, UAF and Total PARCO, Faisalabad, Pakistan, respectively. The selected concentrations of 30 mg kg⁻¹ Cd and 6000 mg kg⁻¹ diesel were used to simulate heavily contaminated industrial soil conditions, as similar co-contamination levels have been reported to significantly influence contaminant bioavailability and remediation efficiency at higher concentrations [53–55]. Following this, each pot was filled with 8 kg of contaminated and uncontaminated soil in respective pots to ensure an adequate supply of nutrients and oxygen for better root growth [56]. Thereafter, the pots were left for 15 days for the aging process in natural field conditions to ensure uniform distribution, stabilization, and interaction of contaminants with the soil matrix [57].

### Experimental layout

The pot experiment was carried out in the research area (31° 26’ 18.3” N, 73°04’ 09.8” E) of ISES, UAF by following a completely randomized design (CRD) for pots arrangement. There were nine treatments, and each was replicated three times. The treatment details and the meteorological data of the experiment are presented in Tables S1 and S3, respectively. The recommended doses of nitrogen (N), phosphorus (P), and potassium (K) were applied at sowing in the form of urea, single super phosphate, and sulphate of potash at the rate of 60, 40, and 25 kg ha^− 1^ into the pots, respectively [58]. The urea was applied in split application during the whole experiment (at sowing, subsequently after 30 days of experiment, and at the flowering stage) [59].

Before sowing the seeds, green-synthesized nZVI particles were incorporated into the pots at a concentration of 1% (v/w). Additionally, a microbial consortium was inoculated at a rate of 25 mL per pot, with a bacterial optical density (OD_600_) of 0.5, corresponding to 10^8^-10^9^ CFU mL^-1^, to ensure uniform distribution and effective interaction with contaminants in the soil matrix. The seeds of sunflower (variety Hysun-33) were obtained from Ayub Agriculture Research Institute (31°24′06.74″ N, 73°03′05.14″ E), Faisalabad, Pakistan and surface sterilized by treating them with 1% sodium hypochlorite (NaOCl) solution for 5 min subsequently rinsed thoroughly with distilled water. Afterwards, the seeds were immersed in distilled water for 10 min [60]. Five seeds were sown per pot, and after germination, two plants were retained in each pot. Tap water was used to maintain 70% field capacity [61], which is considered optimal for sunflower growth and microbial activity in soil, by weighing the pots throughout the experimental period. After 90 days of seed sowing, the plants were harvested, and at that time, various plant and soil attributes were noted, and the degradation of PHCs and Cd contents was evaluated following standard protocols as described below.

### Physicochemical analysis of soil

The physicochemical analysis of normal (uncontaminated) and contaminated soil was carried out before the experiment, and the results are presented in Table S2. The pH and EC were determined by making soil suspensions and dipping the probe in solutions by using a pH meter (HI991003 pH meter, Hanna Instruments, Italy) and an EC meter (HI 99300 EC/TDS meter, Hanna Instruments, Italy), respectively [62]. The 50 g dry soil was mixed with a dispersion solution of sodium-hexa-meta phosphate in a volumetric cylinder. The soil texture was determined by using a hydrometer (ASTM 152 H GILSON Comp Inc., USA) following the protocol of Bouyoucos [63], and saturation percentage was determined by making a saturated paste of soil in accordance with the procedure mentioned by U.S Salinity Laboratory Staff [64]. The N, P, and K contents were analyzed by employing the Kjeldhal apparatus (Behrotest INKJEL K-375, Behr Labor-Technik, Düsseldorf, Germany) in accordance with Bremner and Mulvaney [65], spectrophotometer (VS-1100, Biotechnology Medical Services, China), and flame photometer (Sherwood Model 410, Scientific Limited, Cambridge, UK) followed by Estefan et al. [62], and Chapman and Pratt [66], respectively.

### Seedling emergence and growth parameters of sunflower

The seedling emergence was observed for the first ten days of the experiment until the count of seeds was constant. Following the post-harvest, the length of roots, shoots, and cob diameter (cm) were measured with the help of a measuring tape. The fresh and dry weights of roots, shoots, flowers and total biomass in grams were recorded using a weighing balance. Similarly, the number of leaves, flowers, sepals, petals, and shoots was counted manually [67]. For leaf area (cm^2^), the length and width of leaves of each replicate of a treatment were noted. The leaf area was obtained by multiplying the length and width and calculated by using Eq. 1 [68].

1 $$\:\:\:\:\:\:\:\:\:\:\:\:\:\:\:\:\:\:\:\:\:\:\:\:\:\:\:\:\:\:\:\:\:\:\:\:\:\:\:\:\:\:\:\:\:\:\:\:\:\:\:\:\:A\:\left(cm\right)=L\:\left(cm\right)\:\times\:\:W\:\left(cm\right)\:\:\:\:\:\:\:\:$$

### Physiological and biochemical parameters of sunflower

After 45 days of sowing, a SPAD meter (SPAD-502 Konica, Minolta, Europe) was used to assess the SPAD value of plant leaves. A photosynthetic yield analyzer (MINI-PAM-II) (WALZ Mess und Regeltechnik, Effeltrich, Germany) was employed for the determination of fluorescence yield, photosynthetically active radiation (µmol m^-2^s^-1^), quantum yield, and electron transport rate (mmol m^-2^s^-1^) of each plant [50].

The relative water content (RWC) was determined by the method described by Sairam et al. [69]. A fresh leaf weighing 0.5 g was added in distilled water in the test tube and left for four hours to attain full turgidity. After four hours, the leaf turgid weight was noted with the help of a weighing balance. Following this, the leaf was oven-dried at 68 °C for 48 h, and the dry weight was observed. The RWC was calculated by using Eq. (2).

2 $$\:\:\:\:\:\:\:\:\:\:\:\:\:\:\:\:\:\:\:\:\:\:\:\:\:\:\:\:\:\:\:\:\:\:\:\:\:\:\:\:\:\:\:\:\:\:\:\:\:\:\:RWC\:\left(\%\right)=(FW-DW)/\:(TW-DW)\:\times\:100\:\:\:\:\:$$

The membrane stability index (MSI) was analyzed using the protocol of Sairam et al. [69]. A fresh leaf weighing 0.5 g was dipped in distilled water of test tubes. For the measurement of EC1 and EC2, the test tubes were placed in a water bath at 40 °C for 30 min and subsequently at 100 °C for 10 min, respectively. The MSI was calculated by following Eq. (3).

3 $$\:\:\:\:\:\:\:\:\:\:\:\:\:\:\:\:\:\:\:\:\:\:\:\:\:\:\:\:\:\:\:\:\:\:\:\:\:\:\:\:\:\:\:\:\:\:\:\:\:\:\:\:\:\:\:MSI=\left[\:1-EC1\:/\:EC2\:\right]\:\times\:100\:\:\:\:\:\:\:\:\:\:\:\:\:\:\:\:\:\:\:\:\:$$

The chlorophyll a and b contents were determined by using the protocol of Arnon [70], and carotenoids were measured by using a modified method of Lichtenthaler [71] based on the same absorbance readings of 663 and 645 nm. Briefly, a fresh leaf, weighed 0.5 g, was dipped in 80% acetone (v/v) and left overnight. After that, the samples were run on a spectrophotometer at wavelengths of 663 nm and 645 nm, respectively and calculated by Eqs. 4, 5, and 6, respectively.

4 $$\:\mathrm{C}\mathrm{h}\mathrm{l}\mathrm{o}\mathrm{r}\mathrm{o}\mathrm{p}\mathrm{h}\mathrm{y}\mathrm{l}\mathrm{l}\:\mathrm{a}\:(\mathrm{m}\mathrm{g}/\mathrm{g})=(12.7\:\left(\mathrm{O}\mathrm{D}\:663\right)-2.69\:\left(\mathrm{O}\mathrm{D}\:645\right)\times\:\:V/\:1000\:\times\:W\:\:\:\:\:\:\:\:\:\:\:\:\:\:\:\:\:\:\:\:\:\:\:\:\:$$

5 $$\:\mathrm{C}\mathrm{h}\mathrm{l}\mathrm{o}\mathrm{r}\mathrm{o}\mathrm{p}\mathrm{h}\mathrm{y}\mathrm{l}\mathrm{l}\:\mathrm{b}\:(\mathrm{m}\mathrm{g}/\mathrm{g})=\left(2.29\:\right(\mathrm{O}\mathrm{D}\:645)-4.68\:(\mathrm{O}\mathrm{D}\:663)\times\:\:V/\:1000\:\times\:W\:\:\:\:\:\:\:\:\:\:\:\:\:\:\:\:\:\:\:\:\:\:\:\:\:$$

6 $$\:\mathrm{C}\mathrm{a}\mathrm{r}\mathrm{o}\mathrm{t}\mathrm{e}\mathrm{n}\mathrm{o}\mathrm{i}\mathrm{d}\mathrm{s}\:(\mathrm{m}\mathrm{g}/\mathrm{g})=(20.2\:\left(\mathrm{O}\mathrm{D}\:645\right)+8.02\:(\mathrm{O}\mathrm{D}\:663)\times\:\:V/\:1000\:\times\:W\:\:\:\:\:\:\:\:\:\:\:\:\:\:\:\:\:\:\:\:\:\:\:\:\:\:\:\:$$

Where V is the final volume of the extract (mL) and W is the fresh weight of leaf tissue (g).

### Chemical analysis of plant and soil

#### Cd analysis in plants and soil

The 0.5 g of the ground plant samples were placed in flasks. The concentrated HNO_3_ and HClO_4_ in a ratio of 3:1 were transferred into flasks and allowed to stand overnight. The flasks were placed on a hot plate, and the temperature was increased gradually up to 300 °C under controlled conditions in a fume hood. The plant and soil samples were digested until the color of the solution turned white and grey, respectively. The temperature of the flasks was cooled down, and distilled water (up to 50 mL) was added. The digested samples were filtered with the help of Whatman filter paper [72]. The digested extract was further used for total Cd analysis by atomic absorption spectrophotometer (Hitachi Polarized Zeeman AAS, Z-8200, Japan) [73].

For the determination of bioavailable Cd, a widely accepted DTPA extraction method was used for estimating the plant-available fraction of heavy metals in soils. The 10 g of soil was equilibrated with 20 mL of diethylenetriaminepentaacetic acid (DTPA) extracting solution (0.005 M DTPA, 0.01 M CaCl₂, and 0.1 M triethanolamine, pH 7.3) for 2 h at 25 °C. The suspension was passed through a 100-mesh sieve and filtered through a 0.45 μm membrane filter, and the Cd concentration in the filtrate was quantified using an atomic absorption spectrophotometer [74, 75].

#### Translocation and bioconcentration factors

The translocation factor (TF) and bioconcentration factor (BCF) of metals were determined to assess their translocation from roots to aerial parts and their accumulation in plants by using Eqs. 7 and 8, respectively [72].

7 $$\:TF=\frac{Metal\:accumulation\:in\:shoots\:\left(mg/kg\right)}{Metal\:accumulation\:in\:roots\:\left(mg/kg\right)}\:\:\:\:\:\:\:\:\:\:\:\:\:\:\:\:\:\:\:\:\:\:\:\:\:\:\:\:\:\:\:\:\:\:\:\:\:\:\:\:\:\:\:\:\:\:\:\:\:\:\:\:\:\:\:\:\:\:\:\:$$

8 $$\:BCF=\frac{Metal\:accumulation\:in\:harvested\:plant\:material\:\left(mg/kg\right)}{Metal\:concentration\:in\:soil\:\left(mg/kg\right)}\:\:\:\:\:\:\:\:\:\:\:\:\:\:\:\:\:\:\:$$

#### Analysis of PHCs in soil

For the determination of PHCs in soil, 50 g of soil was mixed with 100 mL of deionized water in a beaker and left overnight [67]. After 24 h, the solution was gently mixed. The analysis of PHCs in soil was performed with the help of a total petroleum hydrocarbon (TPH) analyzer (PHA-100 plus, PETROSENSE^®^, Las Vegas, NV, USA). Briefly, the probe was immersed in the solution for measurement, allowing detection of hydrocarbons present in the liquid phase using infrared (IR) sensing [73]. This approach represents a water-based extraction method, which is primarily used to recover the loosely bound and water-extractable (labile) fraction of hydrocarbons from soil. The precision and accuracy of this reading are ± 10%. Moreover, the analysis focused on TPH and specific intermediate degradation products were not measured.

### Statistical analysis

The least significant differences between the treatments at *p* < 0.05 were observed on the measured data by using analysis of variance based on a simple CRD statistical technique, in Statistix 8.1 software. The relationship among measured attributes was evaluated using the Pearson correlation matrix, visualized through the corrplot package in R software version 4.5.1 [76]. To identify the major source of variation among treatments, principal component analysis (PCA) was conducted using the factoMinerR and factoextra packages [76]. PCA biplots were made to visualize clustering patterns of treatments and the contribution of individual variables to each principal component. The graphical abstract was designed using Canva (www.canva.com).

## Results

### Agronomic attributes of sunflower plants influenced by MC and nZVI application

**Fig. 1:**
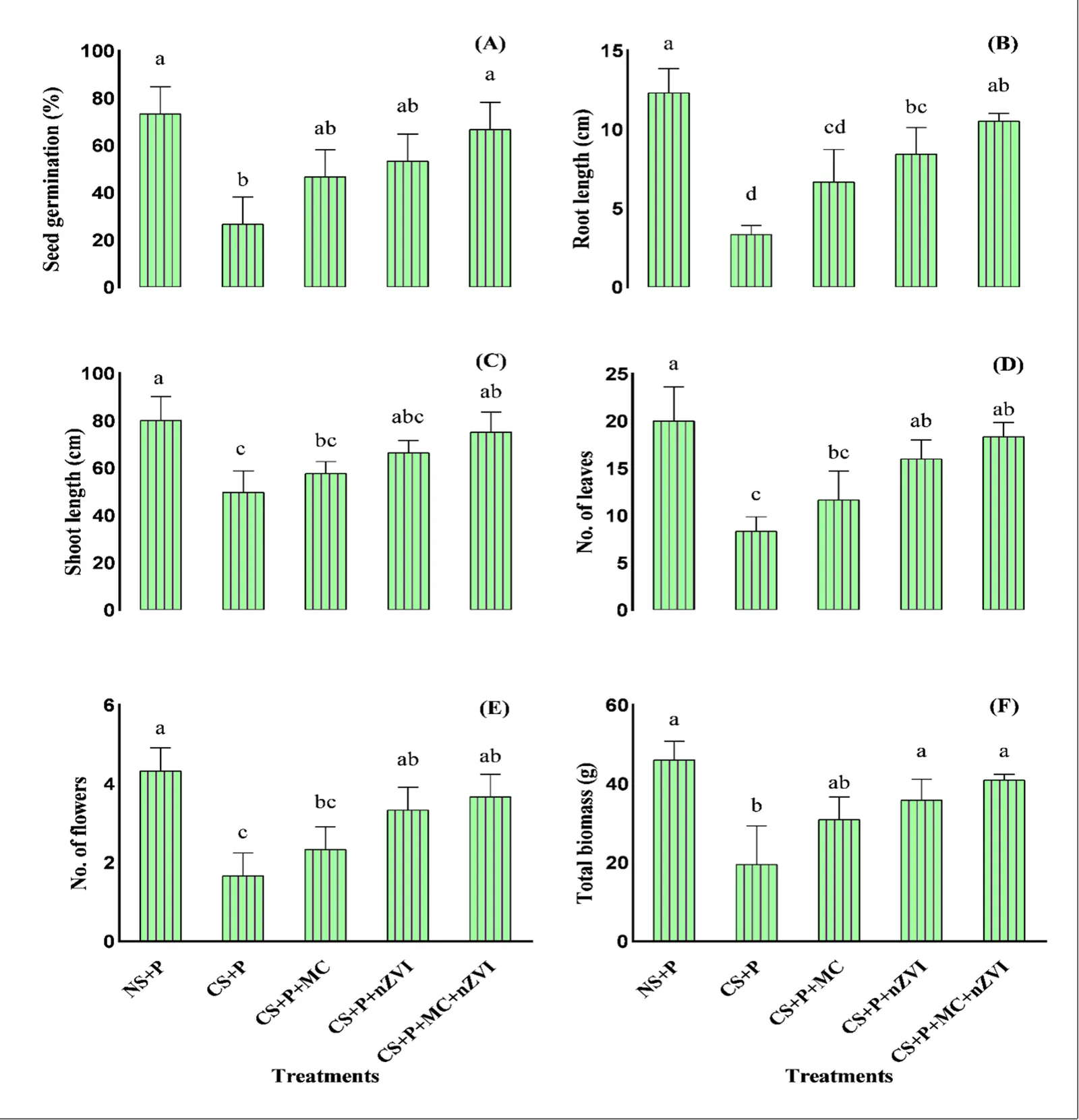
Effects of integrated use of MC and nZVI particles on seed germination (**A**), root (**B**) and shoot (**C**) lengths, number of leaves (**D**), number of flowers (**E**), and total biomass (**F**) of sunflower plants grown in soil co-contaminated with PHCs and Cd. Columns and bars indicate the means and standard deviations, respectively, of triplicate values for different treatments. Different small letters show significant differences among the treatments at *P* ≤ 0.05. NS, Normal (uncontaminated) soil; CS, Contaminated soil; P, Sunflower plant; MC, Microbial consortium; nZVI, Nano zero-valent iron particles

In this study, the co-contamination of PHCs and Cd caused a significant (*p* < 0.05 ) decrease in agronom ic attributes of sunflower, demonstrating 37.8% to 82.5% reduction in seed germination, agronomic and morphological attributes of plants, including root and shoot lengths, root and shoot fresh and dry weights, number of leaves, flowers, sepals, petals, and shoots, total biomass, leaf area, cob diameter, flower fresh and dry weights, as compared to the respective uncontaminated control (Fig. 1; Table 1). While in contaminated treatments where microbial consortium (MC) and nZVI particles were applied individually, there was significant (*p* < 0.05 ) increase in seed germination (42.8% and 50.1%), shoot length (57.7% and 64.4%), shoot fresh weight (26.8% and 29.5%), number of shoots (31.0% and 33.3%), number of sepals (30.6% and 45.0%), number of petals (14.6% and 16.0%), number of leaves (11.6% and 16.0%), number of flowers (28.5% and 50.0%), total biomass (30.9% and 35.8%), leaf area (58.8% and 77.7%), and flower fresh weight (12.5% and 14.2%) of the sunflower, respectively as compared to the respective unamended contaminated control (Fig. 1; Table 1). The combined application of MC and nZVI particles further improved this effect and substantially enhanced the seed germination (60.1%), root (10.5%) and shoot (75.2%) lengths, shoot fresh weight (31.0%), number of shoots (35.6%), number of sepals (50.0%), number of petals (19.0%), number of leaves (18.3%), number of flowers (54.5%), total biomass (41.0%), leaf area (83.5%), and flower fresh weight (16.4%) of the sunflower as compared to the respective unamended contaminated control, demonstrating the significant contribution of microbes and nZVI particles in the enhancement of growth of sunflower plants in the contaminated soil (Fig. 1; Table 1).

**Table 1:** Effects of MC and nZVI particles on morphological attributes of sunflower plants grown in soil co-contaminated with Cd and PHCs

Treatments	RFW (g)	SFW (g)	RDW (g)	SDW (g)	LA (cm^2^)	CD (cm)	NOSh	NOS	NOP	FW (g)	FDW (g)
NS + P	3.92 ± 1.01a	34.3 ± 3.28a	1.87 ± 0.13a	6.10 ± 1.23a	90.0 ± 13.4a	2.80 ± 0.65a	40.0 ± 2.00a	55.0 ± 5.00a	23.0 ± 1.00a	17.4 ± 2.63a	8.64 ± 1.12a
CS + P	1.76 ± 0.38b	17.5 ± 5.91b	0.32 ± 0.18c	2.66 ± 0.46c	27.7 ± 5.02c	1.33 ± 0.40b	19.3 ± 2.51c	20.0 ± 4.00c	6.66 ± 2.88c	8.18 ± 3.26b	4.22 ± 1.25c
CS + P+MC	2.72 ± 0.34ab	26.8 ± 4.57ab	1.21 ± 0.11b	3.96 ± 0.28bc	58.8 ± 6.11b	1.93 ± 0.40ab	31.0 ± 2.64b	30.6 ± 4.04b	14.6 ± 2.08b	12.5 ± 1.41ab	5.32 ± 0.64bc
CS + P+ nZVI	3.14 ± 0.81ab	29.5 ± 7.16ab	1.54 ± 0.09ab	4.58 ± 0.04ab	77.7 ± 6.76ab	2.30 ± 0.10ab	33.3 ± 3.05ab	45.0 ± 3.60a	16.0 ± 1.73b	14.2 ± 3.33ab	6.21 ± 1.53abc
CS + P+MC+ nZVI	3.63 ± 0.26a	31.0 ± 1.56a	1.62 ± 0.22ab	5.57 ± 0.32ab	83.5 ± 11.2a	2.63 ± 0.49a	35.6 ± 2.08ab	50.0 ± 2.00a	19.0 ± 1.00ab	16.4 ± 2.70a	7.33 ± 0.89ab

These parameters of sunflower plants were taken at the time of harvesting (i.e., after 90 days of sowing). The values are means of three replicates ± standard deviation. Means with different letters show significant differences in the treatments at *P* ≤ 0.05

*RFW* Root fresh weight, *RDW* Root dry weight, *SFW* Shoot fresh weight, *SDW* Shoot dry weight, *LA* Leaf area, *CD* Cob diameter, *NOSh* Number of shoots, *NOS* Number of sepals, *NOP* Number of petals, *FW* Flower weight, *FDW* Flower dry weight, *NS* Normal (uncontaminated) soil, *CS* Contaminated soil, *P* Sunflower plants, *MC* Microbial consortium, *nZVI* Nano zero-valent iron particles, *Cd* Cadmium, *PHCs* Petroleum hydrocarbons

### Physiological parameters of sunflower plants influenced by MC and nZVI application

**Fig. 2:**
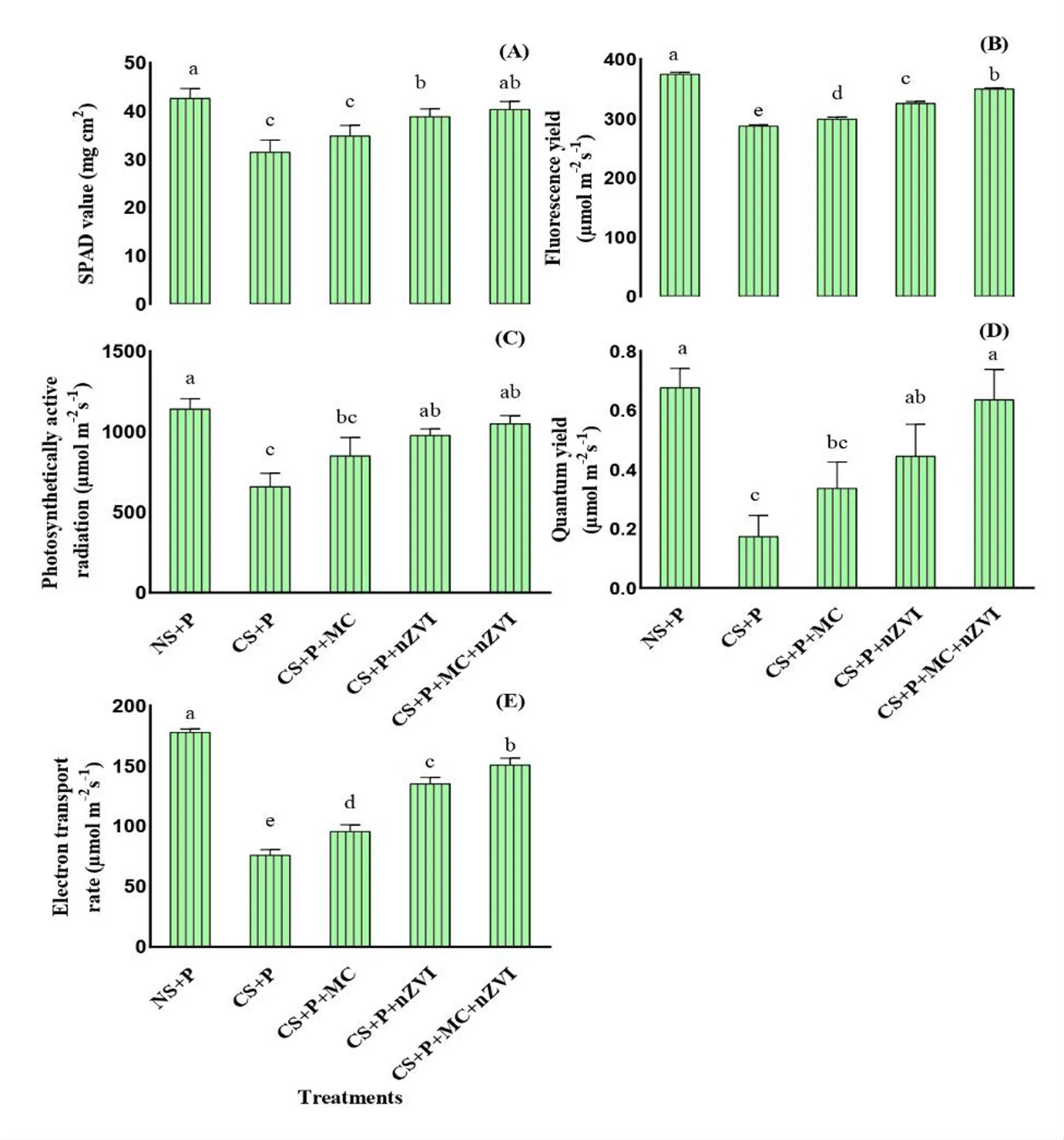
Effects of integrated use of MC and nZVI particles on SPAD value (**A**), fluorescence yield (Ft) (**B**), photosynthetically active radiation (PAR) (**C**), quantum yield (YII) (**D**), and electron transport rate (ETR) (**E**) of sunflower plants grown in soil co-contaminated with PHCs and Cd. Columns and bars indicate the means and standard deviations, respectively, of triplicate values for different treatments. Different small letters show significant differences among the treatments at *P* ≤ 0.05. NS, Normal (uncontaminated) soil; CS, Contaminated soil; P, Sunflower plants; MC, Microbial consortium; nZVI, Nano zero-valent iron particles

The co-contamination with PHCs and Cd also disturbed the physiological parameters of sunflower plants. For example, 23.3%, 42.2%, 74.3%, and 57.2% reduction was observed in fluorescence yield, photosynthetically active radiation, quantum yield, and electron transport rate, respectively, over their respective uncontaminated control (Fig. 2; Table 2). A similar trend of decline in values was observed in the case of SPAD value (26.1%), membrane stability index (11.2%), relative water content (42.5%), chlorophyll a (74.6%), chlorophyll b (59.5%), and carotenoids (64.9%), over their respective uncontaminated control. However, the individual application of MC and nZVI particles enhanced the SPAD value (9.75% and 19.0%), fluorescence yield (3.90% and 11.7%), photosynthetically active radiation (22.4% and 32.5%), quantum yield (48.4% and 61.0%), electron transport rate (20.6% and 43.7%), membrane stability index (8.80% and 25.1%), relative water content (20.0% and 32.5%), chlorophyll a (55.0% and 66.0%), chlorophyll b (37.1% and 54.6%), and carotenoids (33.9% and 56.2%), respectively as compared to their respective unamended contaminated control (Fig. 2; Table 2). Furthermore, the combined additionof MC and nZVI particles had a significant (*p* < 0.05 ) contribution in increasing the SPAD value (21.9%), fluorescence yield (17.9%), photosynthetically active radiation (37.2%), quantum yield (72.7%), electron transport rate (49.6%), membrane stability index (55.2%), relative water content (37.8%), chlorophyll a (70.9%), chlorophyll b (57.7%), and carotenoids (61.3%) as compared to their respective unamended contaminated control (Fig. 2; Table 2).

**Table 2:** Effects of MC and nZVI particles on physiological and biochemical attributes of sunflower plants grown in soil co-contaminated with Cd and PHCs

Treatments	MSI (%)	RWC (%)	Chlorophyll a (mg^− 1^)	Chlorophyll b (mg^− 1^)	Carotenoids (mg^− 1^)
NS + P	38.8 ± 1.60a	94.6 ± 2.58a	0.23 ± 0.02a	0.80 ± 0.04a	0.96 ± 0.02a
CS + P	34.4 ± 3.56ab	54.3 ± 4.50d	0.06 ± 0.04c	0.32 ± 0.03c	0.33 ± 0.04d
CS + P+MC	31.4 ± 2.43ab	68.0 ± 5.56c	0.13 ± 0.03bc	0.52 ± 0.04b	0.51 ± 0.03c
CS + P+ nZVI	25.8 ± 4.61bc	80.5 ± 5.35b	0.17 ± 0.03ab	0.72 ± 0.03a	0.77 ± 0.04b
CS + P+MC+ nZVI	15.4 ± 8.10c	87.4 ± 4.47ab	0.20 ± 0.02ab	0.77 ± 0.02a	0.87 ± 0.05ab

The values are means of three replicates ± standard deviations. Means with different letters show significant differences in the treatments at *P* ≤ 0.05

*MSI* Membrane stability index, *RWC* Relative water content, *NS* Normal (uncontaminated) soil, *CS* Contaminated soil, *P* Sunflower plants, *MC* Microbial consortium, *nZVI* Nano zero-valent iron particles, *Cd* Cadmium, *PHCs* Petroleum hydrocarbons

### Soil nutrients and their uptake by sunflower plants influenced by MC and nZVI application

**Fig. 3:**
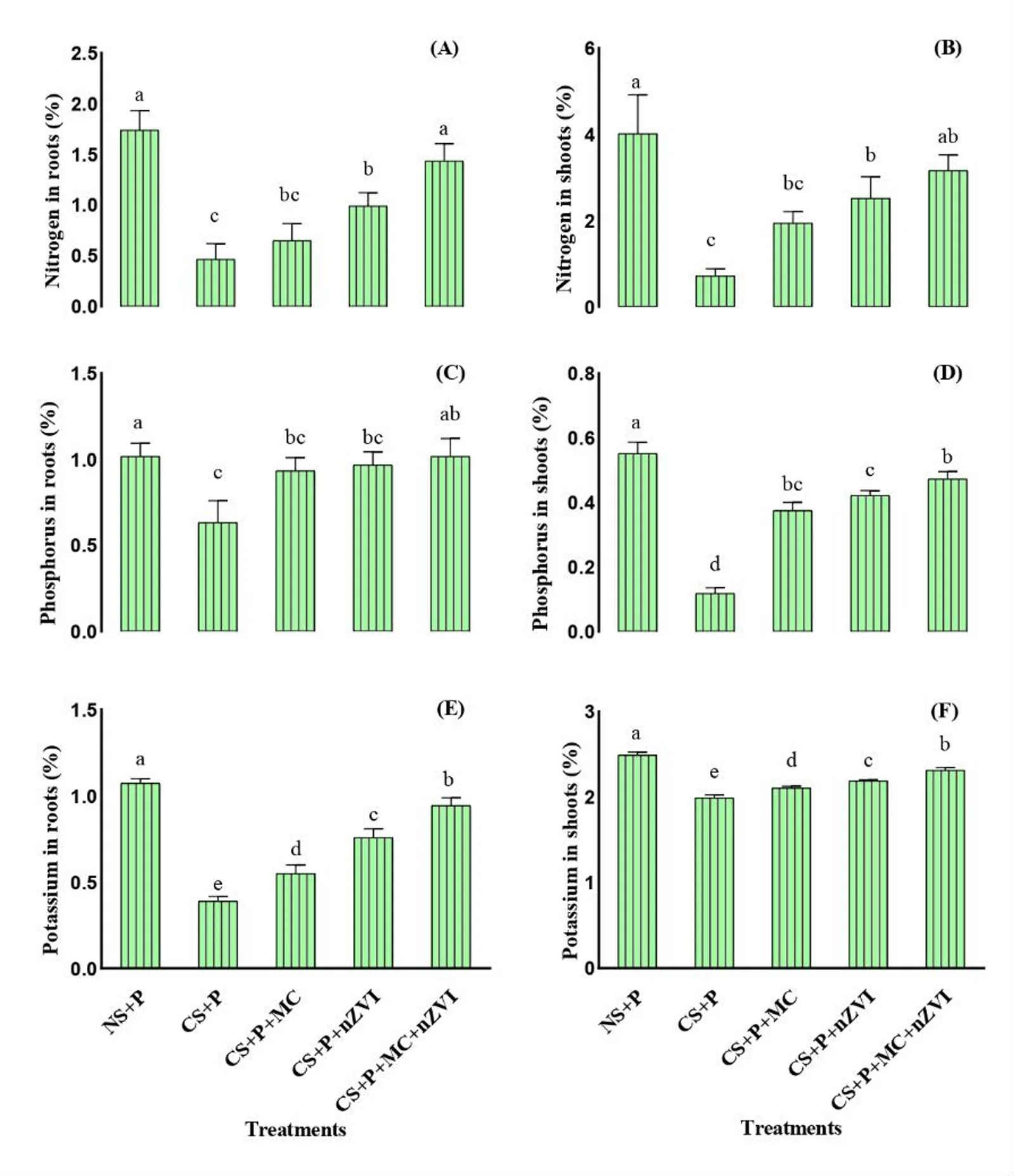
Effects of integrated use of MC and nZVI particles on acquisition of nitrogen in roots (**A**), nitrogen in shoots (**B**), phosphorus in roots (**C**), phosphorus in shoots (**D**), potassium in roots (**E**), and potassium in shoots (**F**) of sunflower grown in soil co-contaminated with PHCs and Cd. Columns and bars indicate the means and standard deviations, respectively, of triplicate values for different treatments. Different small letters show significant differences among the treatments at *P* ≤ 0.05. NS, Normal (uncontaminated) soil; CS, Contaminated soil; P, Sunflower plants; MC, Microbial consortium; nZVI, Nano zero-valent iron particles

The results revealed a substantial reduction in N, P, and K accumulations in roots (73.2%, 37.7%, and 63.6%) and shoots (82.1%, 78.5%, and 20.0%) of sunflower plants, respectively, under Cd and PHC co-contamination as compared to the respective uncontaminated control (Fig. 3). However, the individual application of the MC significantly improved nutrient acquisition in roots (28.3% N, 32.1% P, and 29.0% K) and shoots (63.1% N, 68.4% P, and 5.53% K) when compared to the respective uninoculated contaminated control. Likewise, individual nZVI particle application also resulted in an increase of N, P, and K contents in roots up to 53.0%, 34.4%, and 48.4%, and in shoots up to 71.6%, 71.9%, and 9.13%, respectively, over the respective control (Fig. 3). The combined application of MC and nZVI particles further amplified N, P, and K uptake with an increase of 67.5%, 37.7%, and 58.6% in roots and 77.3%, 75.0%, and 13.9% in shoots, respectively, as compared to the unamended control treatments, suggesting their significant (*p* < 0.05 ) role in enhancing nutrient uptake by plants in contaminated soil (Fig. 3).

**Fig. 4:**
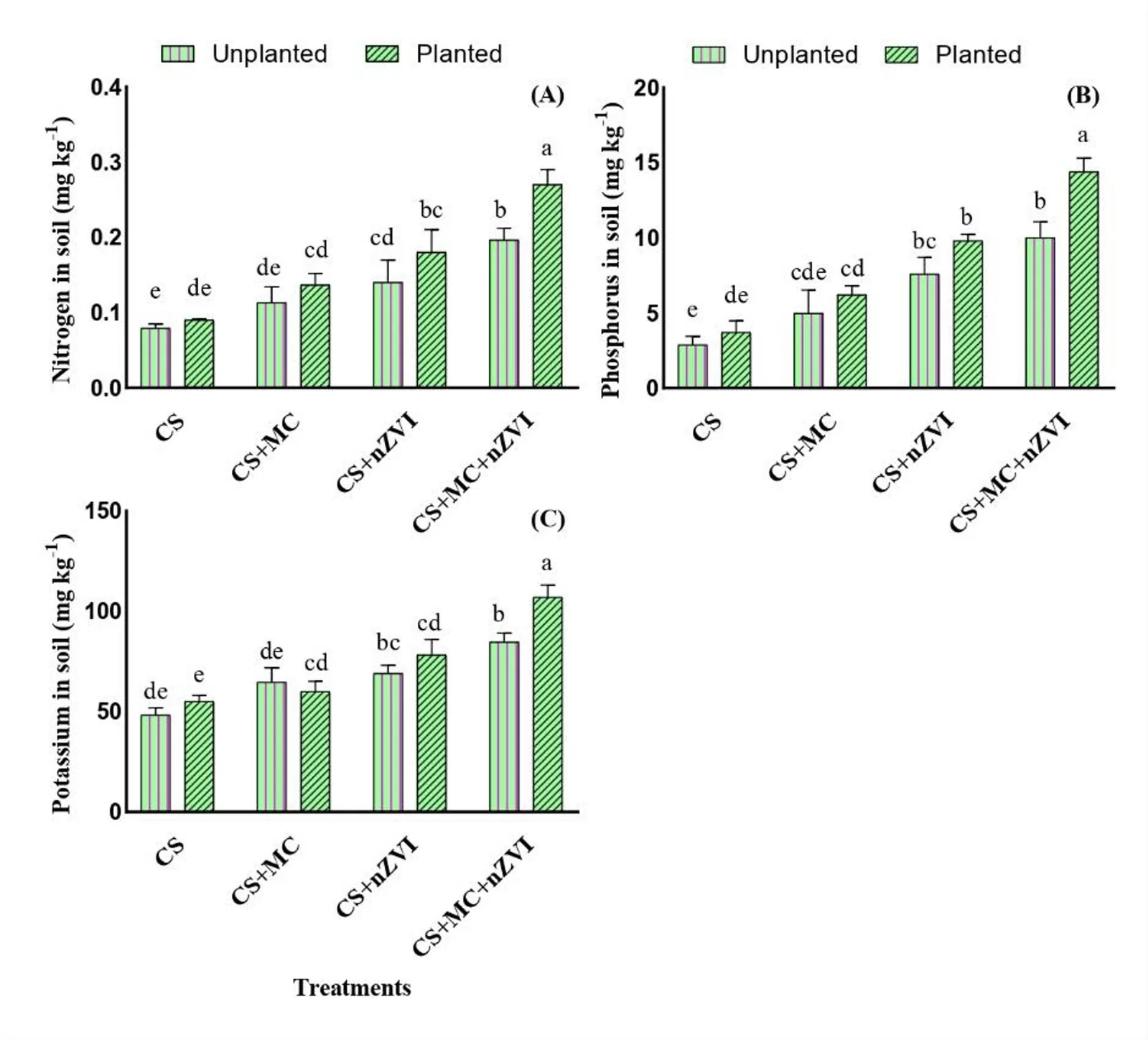
Effects of integrated use of MC and nZVI particles on nitrogen (**A**), phosphorus (**B**), and potassium (**C**) in soil co-contaminated with PHCs and Cd. Columns and bars indicate the means and standard deviations, respectively, of triplicate values for different treatments. Different small letters show significant differences among the treatments at *P* ≤ 0.05. NS, Normal (uncontaminated) soil; CS, Contaminated soil; P, Sunflower plants; MC, Microbial consortium; nZVI, Nano zero-valent iron particles

Interestingly, regardless of contamination and amendments, the N, P, and K content in soil remained higher in planted treatments over the respective unplanted treatments (Fig. 4). For example, N, P, and K contents were 11.8%, 22.5%, and 12.1%, lower in unplanted and unamended treatments than their counterparts. However, regardless of vegetation, addition of MC and nZVI significantly enhanced nutrient levels in soil (Fig. 3b). The individual application of MC in the planted treatment enhanced the N, P, and K contents in soil by 17.0%, 19.8%, and 7.20%, respectively. Similarly, when nZVI particles were applied individually, there was also an increase in N, P, and K values up to 22.2%, 22.4%, and 11.9%, respectively, over their respective counterparts (Fig. 4). Moreover, the combined application of MC and nZVI particles further enhanced the N, P, and K contents (up to 30.5%) in soil, indicating the substantial synergistic impacts of microbes and nZVI particles in improving nutrient levels in contaminated soil (Fig. 4).

### Remediation of Cd and PHCs from soil and Cd accumulation in plant tissues

**Fig. 5:**
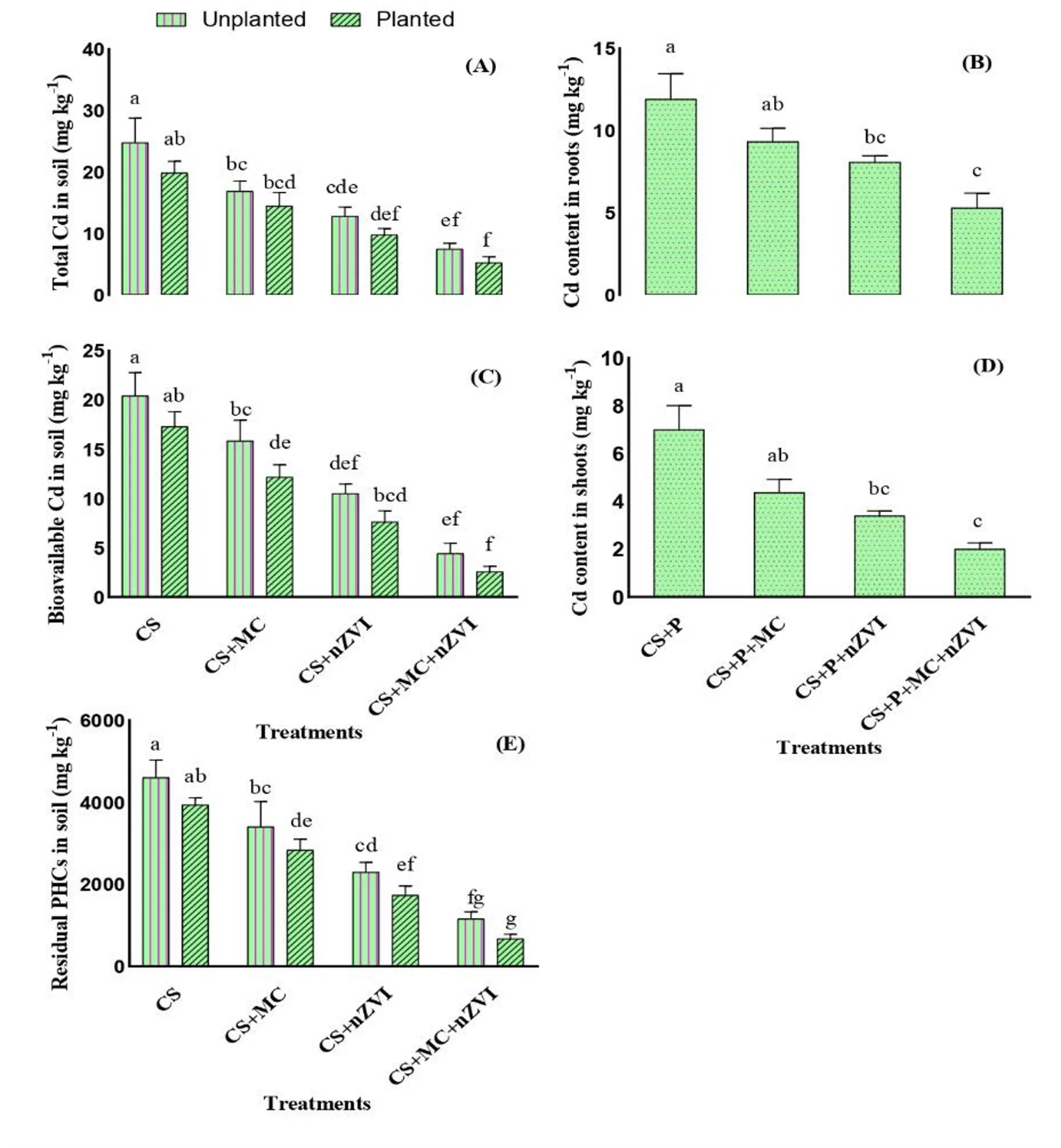
Effects of integrated use of MC and nZVI particles on removal of total cadmium from soil (**A**), residual cadmium in roots of sunflower plants (**B**), bioavailable cadmium in soil (**C**), residual cadmium in shoots of sunflower plants (**D**), and petroleum hydrocarbons in soil (**E**) co-contaminated with PHCs and Cd. Columns and bars indicate the means and standard deviations, respectively, of triplicate values for different treatments. Different small letters show significant differences among the treatments at *P* ≤ 0.05. CS, Contaminated soil; P, Sunflower plants; MC, Microbial consortium; nZVI, Nano zero-valent iron particles

In this study, the maximum uptake and accumulation of Cd in root and shoot tissues of sunflower plants was observed in the unamended contaminated control treatment (Fig. 5b and c). However, by the individual application of MC and nZVI particles, a significant (*p* < 0.05 ) decrease was observed in Cd uptake in both roots (21.6% and 32.1%) and shoots (37.6% and 51.4%), respectively, when compared to the respective unamended treatment. Furthermore, the integrated treatment of MC and nZVI particles performed best in reducing the accumulation of Cd in roots (55.5%) and shoots (71.4%) of sunflower plants (Fig. 5b and c), suggesting a significant synergistic role of MC and nZVI particles in minimizing the metals uptake by plants and their subsequent entry in the food chain.

In this study, the bioavailable fraction of Cd was also calculated. The results showed a higher amount of bioavailable concentration of Cd in the unamended treatments as compared to the respective control (Fig. 5c). While the treatment with individual application of MC and nZVI particles in planted treatment decreased the bioavailability of Cd up to 23.1% and 27.3%, respectively, as compared to the respective unamended control. Additionally, the integrated application of MC and nZVI particles performed best and further reduced the bioavailable Cd (42.1%) in soil in comparison to the respective counterparts (Fig. 5c).

After 90 days of experiment, 23.3% and 17.4% reduction in residual concentrations of PHCs and Cd, respectively, was observed in the unamended unplanted control as compared to their initially applied concentrations (Fig. 5a and e). Based on the residual concentrations of PHCs and Cd, plants alone phytoremediated up to 34.3% PHCs and 33.7% Cd as compared to their initially applied levels in soil, demonstrating up to 19.7% higher pollutant removal by plants over their unplanted counterparts (Fig. 5a and e). In the unplanted soil, the individual application of MC and nZVI particles revealed substantial removal of PHCs and reduction in Cd as compared to their respective unamended counterparts (Fig. 5a and e). For instance, nZVI particles alone demonstrated 50.0% and 48.3% higher removal of PHCs and Cd, respectively, than their unamended unplanted control. Quite a similar trend of individual application of nZVI particles and MC was observed in the case of planted treatments (Fig. 5a and e). The efficacy of nZVI particles and MC for pollutant removal was further enhanced in their combined application, showing the highest remediation (up to 82.7%) of both pollutants among all treatments. In general, regardless of MC and nZVI particles, planted treatments showed higher removal of both pollutants than their unplanted counterparts (Fig. 5a and e). Moreover, overall, both nZVI particles and MC were more effective in the remediation of PHCs than Cd (Fig. 5e), suggesting the higher efficacy of this remedial approach for organic pollutants compared to heavy metals.

### Translocation and bioconcentration factor of Cd

**Fig. 6:**
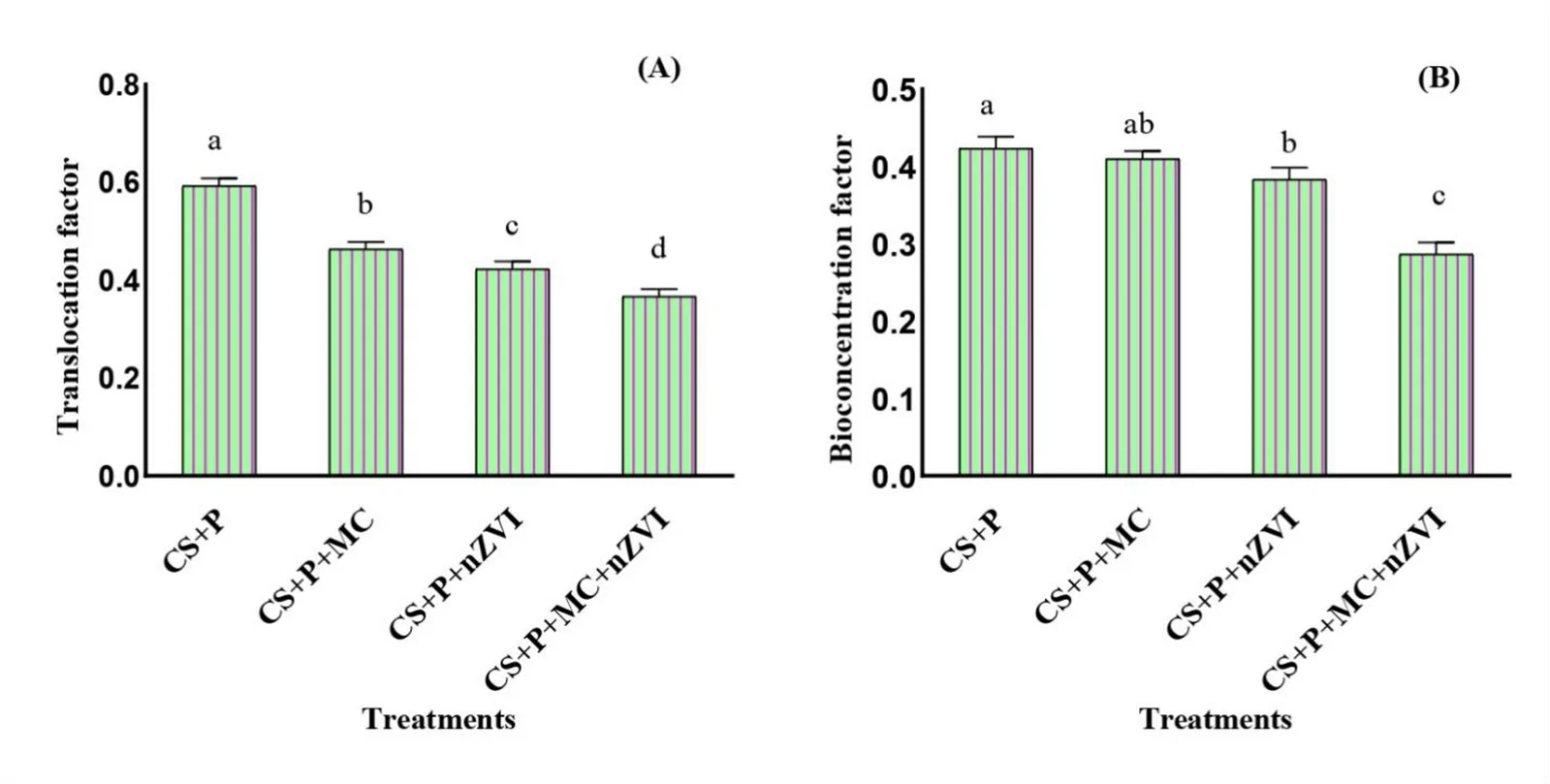
Effects of integrated use of MC and nZVI particles on translocation (**A**) and bioconcentration (**B**) factors in sunflower plants grown in soil co-contaminated with PHCs and Cd. Columns and bars indicate the means and standard deviations of triplicate values for different treatments. Different small letters show significant differences among the treatments at *P* ≤ 0.05. CS, Contaminated soil; P, Sunflower plants; MC, Microbial consortium; nZVI, Nano zero-valent iron particles

The translocation factor (TF) of Cd was highest in the unamended contaminated soil treatment (0.58), indicating significant Cd movement from roots to shoots (Fig. 6a). The lower TF value (less than 1) revealed that the sunflower plant is not a hyperaccumulator for Cd. The translocation of Cd from root to shoots was slightly decreased by the addition of MC (0.46) and nZVI (0.42) particles. The combined application of MC and nZVI particles further reduced the TF value (0.37) (Fig. 6a) and thus movement of Cd from roots to shoots, demonstrating an effective synergistic role of MC and nZVI particles in inhibiting the entry of pollutants into the food chain. A similar trend was observed in bioconcentration factor (BCF), indicating that sunflower had a limited capacity to accumulate Cd relative to its concentration in the soil. Overall, the patterns of BCF (< 1) and TF (< 1) suggest that a sunflower plant in combination with MC and nZVI particles mainly acted as a phytostabilizer (Fig. 6b), effectively immobilizing Cd in the root zone and reducing its upward movement to the shoots.

### Correlation and principal component analysis

The correlation matrices showed pairwise correlation among agronomic, physiological, and biochemical attributes (Figure S2). The Cd in shoots, roots, and soil, and PHCs in soil, exhibited a moderate to strong negative correlation with agronomic, physiological, and biochemical attributes of sunflower plants. Overall, sunflower growth, phyiological and biochemical attributes were strongly positively correlated (*r* > 0.95), whereas Cd and PHCs (*r* = -0.97 to -0.001) showed an antagonistic relationship with agronomic, physiological and biochemical attributes ranging from (-0.97 to -0.001), reflecting detrimental impacts of Cd and PHCs contamination on plant physiology and growth.

Principal component analysis was also performed to examine the relationship among agronomic, physiological, and biochemical attributes of plants and soil under co-contamination of PHCs and Cd (Figure S3). The first two principal components, PC1 and PC2, explained over 90% of the total variance, indicating that these components strongly show the variation in the dataset (Figure S3). In the treatment score plot (A), unamended contaminated treatments such as CS + MC + nZVI, CS + MC, and CS + nZVI were distinctively separated from contaminated treatment (CS) along Dim1, suggesting that these interventions induced marked changes in plant traits. The treatments on the positive side of Dim1 showed a positive correlation with higher growth and favorable physiological profiles, while treatments like CS and CS + P on the negative side revealed higher stress indicators and reduced function. A strong association with plant health was indicated by the variable plot (B), showing strong alignment of agronomic and physiological traits across Dim1. As compared to this, an inverse relationship to plant health was revealed by variables associated with contaminants such as Cd and PHCs in soil and Cd in roots and shoots pointed along Dim1, under co-contaminated soil (Figure S3). Overall, the PCA highlights how treatment effects and stress responses are reflected collectively in agronomic, physiological, and biochemical variables, allowing for clear differentiation of various treatment impacts and underlying plant health status.

## Discussion

The high toxicity and persistence of Cd and PHCs in co-contaminated soils pose a serious environmental threat to soil quality and plant performance [77]. Here we evaluated the individual and combined effect of nZVI particles and microbial consortium on soil remediation and growth of sunflower plants in soil co-contaminated with PHCs and Cd. Seed germination, plant growth, physiological processes, and nutrient acquisition by the sunflower plants were severely affected by soil contamination with Cd and PHCs; however, the co-application of nZVI particles and microbial consortium significantly alleviated the adverse effects of pollutants on sunflower plants and improved the pollutant remediation by metal immobilization and PHCs degradation.

### Impacts of MC and nZVI particles on the seed germination and growth attributes of sunflower plants

Seed germination is an important aspect of the plant life cycle that directly affects the seedling establishment and subsequent crop productivity [78]. In this study, strong inhibitory effects of pollutants were highlighted by a distinctly reduced germination rate of sunflower seeds in co-contaminated soil. The hydrophobic nature of PHCs likely contributed to this reduction by restricting gas exchange and water uptake by the formation of physical barriers on seed coats [79]. This limitation can further suppress germination by delaying the enzymatic activation required for starch mobilization and respiratory activity [80]. Alongside PHCs, Cd also inhibited cell division by interfering with early developmental processes and disrupting the mitotic activity in the embryonic axis, as a phytotoxic metal during seedling emergence [81]. The germination rate was significantly enhanced by the integrated application of nZVI particles and microbial consortium under co-contamination of Cd and PHCs. This enhancement may be attributed to the ability of nZVI particles that reduced contaminant bioavailability through adsorption and redox-mediated reactions, thereby lowering the associated phytotoxic stress [82]. Meanwhile, plant growth-promoting substances, such as siderophores, gibberellins, and indole-3-acetic acid production by the added microbial consortium, could support plant vigor during germination that aids in metabolic activation and cell differentiation [83].

The morphological traits of plants, such as root and shoot lengths and biomass, are the direct indicators of plant health and productivity [84]. This study indicated a substantial reduction in fresh and dry biomasses, accompanied by a decline in root and shoot elongation in sunflower plants grown in soil co-contaminated with PHCs and Cd. The possible explanation behind these undesirable effects is that the PHCs limit oxygen diffusion and aeration in roots by blocking soil porosity and pore spaces, as a result inhibiting root elongation and plant respiration [26]. This stress was further amplified by Cd, which severely interfered with the absorption and translocation of essential nutrients (Fig. 3), vital for structural development, chlorophyll synthesis, and enzymatic activity [85]. This dual stress became apparent as chlorosis, necrosis, and pronounced growth inhibition in plants observed in this study [86]. However, significant morphological improvements in plants were observed in the combined approach of nZVI particles and microbes. A dual role was played by nZVI particles in immobilizing Cd through adsorption and transformation in less bioavailable forms and by degrading hydrocarbon fractions through redox reactions [87]. Meanwhile, plant growth-promoting microbes in the consortium released plant growth-promoting metabolites that supported cellular differentiation and nutrient uptake and also reduced phytotoxicity by degrading hydrocarbons through biosurfactant production [88]. Furthermore, healthier root and shoot development and improved biomass accumulation could be supported by reduced phytotoxicity due to the synergistic interactions of nZVI and microbes, restoring plant growth under co-contamination. Based on the findings, it is stated that the negative impacts of Cd and PHCs can be effectively reduced by the integrated application of nZVI particles and beneficial microbes, which also helps in the restoration of early growth processes, successful plant establishment, and improved plant productivity.

### Impacts of MC and nZVI particles on plant physiology and nutrient acquisition

A significant decline in physiological traits was observed in sunflower plants under PHCs-Cd co-contamination. This impact can be explained by the fact that both hydrocarbons and metals cause the generation of reactive oxygen species (ROS), resulting in a substantial decline in chlorophyll content and overall plant physiology. It has been well-established that ROS cause damage to chloroplast membranes and chlorophyll pigments, which are the common indicators of oxidative stress [89]. The disruption of photosynthesis could be further enhanced by Cd, which interferes with chloroplast ultrastructure and inhibits key enzymes such as Rubisco and chlorophyll synthase in plants [90]. These physiological impairments ultimately restrict plant growth by collectively compromising carbon fixation, water retention, and turgor maintenance in plants [91]. The decline in plant physiology could be attributed to a weaker energy conversion efficiency and reduced chlorophyll fluorescence parameters, such as fluorescence yield, electron transport rate, quantum yield, and photosynthetic active radiation observed in this study [92]. On the other hand, the combined use of nZVI and microbes led to considerable recovery in the physiological performance of sunflower plants under co-contamination of PHCs and Cd. This could be explained by the fact that the nZVI particles contribute to mitigating ROS accumulation in the system and stabilizing cellular structures, while microbial mechanisms enhance osmotic balance and nutrient availability to plants by osmolyte synthesis, organic acid secretion, and siderophore production [93]. In addition to plant’s antioxidant enzymes, the mitigation of oxidative stress may also be partly attributed to the residual phytochemicals (e.g., polyphenols and flavonoids) present on the surface of green-synthesized nZVI. These capping agents possess inherent antioxidant properties that can scavenge ROS, thereby contributing to cellular protection and improved plant physiological performance under stress conditions. Moreover, the photosynthetic activity and stress tolerance are further strengthened by phytohormones produced by the microbes [94]. Thus, it can be stated that the photosynthetic and physiological processes hampered by the contaminants could effectively be restored by nZVI-microbe integration.

A fundamental part of plant metabolism, structural integrity, and enzyme activation is the uptake and transport of essential nutrients such as N, P, K, Mg, Zn, Ca, and Fe in plants [95]. Cd, as a dominant inhibitory factor under co-contaminated conditions, severely reduced nutrient uptake in this study. It is known that Cd competes directly with essential cations at root transporter proteins, often replacing or blocking ions such as Ca²⁺, Zn²⁺, and Fe²⁺, and it also inhibits proton-pumping ATPases that maintain the electrochemical gradient necessary for nutrient absorption [96, 97]. An additional layer of stress was further added by the addition of PHCs, creating oxygen-poor microsites and altering rhizosphere microbial communities, which could further diminish nutrient mobilization [98]. However, interestingly, in this study, these effects were substantially reversed by the integrated treatment of microbes and nZVI particles. More likely, due to the reason that the nZVI particles could facilitate better nutrient uptake and retention, reduce Cd-induced oxidative membrane damage, improve soil structure, and increase the soil cation exchange capacity [99]. Moreover, the microbes in the applied consortium further contributed to this effect by three mechanisms that directly enhance nutrient acquisition, including (i) siderophores production that chelate Fe, (ii) atmospheric nitrogen fixation, and (iii) phosphate solubilization [100]. This suggests that co-use of nanoparticles and microbes could be effective in restoring nutrient dynamics disrupted by the toxic contaminants and making the plant system more supportive and nutritionally healthy. It also indicates the importance of synergies between microbes and nZVI particles for the physiological improvement in sunflower plants.

### Impact of MC and nZVI particles on Cd immobilization and PHCs degradation

Effective, reliable and eco-friendly integrated approaches are needed for the success of any remediation systems and treatment of soil contaminated with multiple contaminants [101]. In this study, the integrated use of nZVI and microbes was very effective in removing significant amounts of PHCs and immobilizing Cd in the soil. It has been reported that the nZVI particles contribute as a powerful reducing agent [31], by converting long-chain hydrocarbons into simpler and less toxic compounds and with the breakdown of PHCs molecules through electron transfer reactions. In the case of metal, nZVI particles demonstrated their strongest impact on Cd immobilization by drastically lowering its bioavailability through reducing mobile Cd²⁺ to less soluble species such as Cd(0) and Cd(OH)₂ [102, 103]. This reduction step is particularly important because Cd mobility directly governs toxicity levels, translocation to shoots, and entry into the food chain. The reduced TF under nZVI treatments might be attributed to Cd immobilization in the rhizosphere via surface interactions with iron nanoparticles. Upon oxidation, nZVI forms iron oxide/hydroxide layers with reactive functional groups that adsorb and complex Cd^2+^ ions, reducing their mobility and bioavailability. This limits Cd uptake by roots and restricts its transport to the xylem, thereby decreasing its translocation from roots to shoots. Overall, nZVI particles demonstrated significant potential to reduce Cd uptake in sunflower plants, which may be attributed to the metal-binding properties of the synthesized nanoparticles. The surface characteristics of nZVI, including its porous morphology and nanoscale size (as observed in SEM analysis), together with the presence of surface functional groups identified through FTIR spectroscopy, may provide reactive sites for chemisorption and electrostatic interactions with Cd ions. These combined properties likely enhance the immobilization of heavy metals in the soil–plant system (for detailed characterization see Supplementary Information) [34, 103]. Furthermore, nZVI particles can also adsorb and immobilize Cd by surface complexation, ion exchange, and precipitation [35], resulting in a reduction in bioavailable fraction in the plant rhizosphere, limiting plant acquisition and further translocation of Cd in plant tissues. While the synthesized nanoparticles were characterized for morphology, crystallinity, functional groups, and particle size distribution, zeta potential measurements were not performed and therefore, the colloidal stability and aggregation behavior of the nanoparticles could not be fully assessed. To address this limitation, future investigations alongside field-scale validation and molecular characterization of degradation pathways and associated genes are suggested.

On the other hand, microbes could use multiple Cd sequestration mechanisms, including biosorption, intracellular accumulation, and extracellular precipitation with enzymatic degradation of hydrocarbons via oxygenases, peroxidases, and dehydrogenases [81]. The co-metabolism mechanism has been reported by certain strains used in this study, such as *Achromobacter xylosoxidans*, *Stenotrophomonas* sp., and *Stenotrophomonas rhizophila*, which were selected because of their demonstrated hydrocarbon-degrading potential and plant growth-promoting traits, including tolerance to contaminated environments, which make them suitable for treating soil co-contaminated with Cd and PHCs, and survive under multiple pollutant stress [25].

Natural attenuation of PHCs and Cd immobilization in unamended contaminated treatment observed in this study is consistent with findings of González-Moscoso et al. [104] and Kumar et al. [105], reporting substantial removal (25–30%) of petroleum hydrocarbons under the natural attenuation process over 240 days in soil. The natural attenuation may include biodegradation by native microorganisms, abiotic transformation (photodegradation, chemical oxidation), and sorption of pollutants to soil particles [28]. In the case of Cd, the metal reduced extractability under natural attenuation could be due to sorption and immobilization of Cd in the soil matrix [105].

In this study, the nZVI-microbes mediated remediation process was further enhanced by the co-existence of sunflower plants. The plant rhizosphere provides essential nutrients and oxygen to pollutant-degrading aerobic microbes, and in return, microbes harboring plant growth-promoting characteristics such as synthesis of organic acids, chemical transformation, siderophore formation, nitrogen fixation and phosphate solubilization, enhance overall physiology, nutrient uptake, growth and phytoremediation ability of plants in contaminated soil [27, 67].

Moreover, the reduced translocation and bioconcentration factors for Cd observed under the combined treatment of microbes-nanoparticles-plants suggest that the tested integrated approach is very effective and useful in reducing the entry of toxic pollutants into the food chain [25, 50]. Overall, the synergistic application of nZVI particles and microbial consortium created a less hazardous environment for plant establishment in soil co-contaminated with organic and inorganic pollutants and facilitated the remediation process by immobilizing toxic metals, degrading hydrocarbons, and minimizing their entry into the plant tissues. Moreover, the synergy between the microbial consortium and nZVI may also be linked to iron-mediated microbial metabolism. During remediation, nZVI is progressively oxidized to produce Fe²⁺/Fe³⁺ oxides or hydroxides that can act as redox-active surfaces and potential electron acceptors for microorganisms. Such Fe-mediated electron transfer may support microbial respiration and accelerate hydrocarbon transformation, especially under oxygen-limited microsites in contaminated soil.

### Implications and future recommendations

This study reports and highlights the significant potential of nano-enhanced bioremediation as a versatile approach for restoring soils contaminated with both organic and inorganic contaminants. Here, we created a resilient biosystem by combining beneficial microbes and green-synthesized nZVI particles that concurrently improved plant productivity, mitigated toxic metal uptake in plants, enhanced pollutant degradation, and ameliorated the adverse effects of mixed contamination. Based on the observed improvements in plant growth, seed germination, nutrient uptake, physiological functioning and pollutant breakdown, we suggest that this multifaceted approach with a highly collaborative system and wide environmental benefits would be very effective and useful in improving agricultural productivity in soil contaminated with multiple pollutants. The findings from this study also contribute to addressing multiple SDGs, including 2 (Zero Hunger), 3 (Good Health and Well-Being), and 15 (Life on Land), by improving agricultural productivity and food safety, and restoring contaminated soils and preventing pollutant transfer through a green, cost-effective, and sustainable remediation strategy. For future work, this microbes-nanoparticles strategy should be tested with various plant species to focus on the long-term stability under field conditions and long-term ecological and financial impacts to ensure practical applicability, and possible improvement of nanoparticle dosage should also be investigated in the future. Moreover, various pollutant and microbial attributes such as PHCs degradation intermediates, soil microbial activity, including basal respiration or microbial biomass, and post-harvest evaluation of inoculated microbial strains should also be considered before applying this technology on field scale remediation work. A cost-benefit analysis of this integrated bioremediation strategy would be essential to evaluate the economic feasibility of the tested approach and the sustainability of the proposed system. Furthermore, deeper molecular insights into the fundamental mechanisms and understanding of their interactions with the soil-plant-microbiome are also crucial for practical implications and interpretation of impacts.

## Conclusions

In this study, we tested and demonstrated the effectiveness of nano-enhanced bioremediation in improving plant growth and remediating soil co-contaminated with PHCs and Cd. A considerable reduction in contaminant-induced stress and improvement in plant growth, physiological, and biochemical traits were observed by the co-use of nZVI particles and microbes. The co-application of green-synthesized nZVI particles and a microbial consortium with sunflower plants successfully remediated both pollutants from soil, where PHCs were mainly degraded by microbes-assisted rhizoremediation, and Cd was immobilized by nZVI-mediated phytostabilization, hindering plant uptake and minimizing entry of toxic metal into food chains. In short, this integrated multifaceted approach offers a promising and sustainable strategy for restoring soil co-contaminated with organic and inorganic pollutants and improving agricultural productivity under multiple pollutant stress.

## Supplementary Information

Supplementary Material 1: Table S1. Treatment details of this study. Table S2. Physicochemical analysis of normal and contaminated soil used in this study. Table S3. Average meteorological data (minimum-maximum) of the experiment time (3 months). Figure S1. SEM images of green-synthesized nZVI particles showing their spherical morphology and uniform distribution (a), FTIR spectrum of nZVI particles showing functional groups associated with plant extract capping and stabilization (b), XRD pattern of nZVI particles confirming the presence of zero-valent iron peaks (c), and zeta sizer analysis of green-synthesized nZVI particles showing the hydrodynamic diameter and size distribution profile (d). Figure S2. Pearson correlation matrix among growth, physiological, and biochemical attributes of sunflower plants grown in soil co-contaminated with PHCs and Cd. Figure S3. Principal component analysis (PCA) biplot showing the distribution of treatment combination, color indicates the quality of representation (cos2) (A), and contribution of different attributes (B), while in the attributes plot, color reflects the contribution of each attribute in PCA. Characterization of nZVI particles.

## Data Availability

The original contributions presented in this study are included in the article and/or supplementary material, further inquiries can be directed to the corresponding author.

## Abbreviations

Cd: Cadmium
PHCs: Petroleum hydrocarbons
nZVI: Nano zero-valent iron
MC: Microbial consortium
YII: Photochemical quantum yield
Ft: Fluorescence yield
PAR: Photosynthetically active radiation
ETR: Electron transport rate
MSI: Membrane stability index
RWC: Relative water content
FW: Fresh weight
TW: Turgid weight
DW: Dry weight
PCA: Principal component analyses
OD: Optical density
TF: Translocation factor
BCF: Bioconcentration factor
SEM: Scanning electron microscope
FTIR: Fourier transform infrared spectroscopy
XRD: X-ray diffraction
