# Supplementary material for: Nano-enhanced bioremediation of soil co-contaminated with petroleum hydrocarbons and cadmium using Helianthus annuus L. plants
Source: BMC Plant Biol. 2026 Aug 13;26:1925. doi: 10.1186/s12870-026-09746-6 (PMC13749690; doi:10.1186/s12870-026-09746-6)
Supplement: Supplementary file 1 [file 12870_2026_9746_MOESM1_ESM.docx]

**Nano-enhanced bioremediation of soil co-contaminated with petroleum hydrocarbons and cadmium using *Helianthus annuus* L. plants**

Visha Habib^1^, Muhammad Imran Khan^1,*^, Qammar Farooq^1^, Muhammad Hayder Ali^1,2^, Hafiz Naeem Asghar^1^, Muhammad Naveed^1^, Majed Alotaibi^3^, Nawab Ali^4^, Mahmoud F. Seleiman^3^

^1^Institute of Soil and Environmental Sciences, University of Agriculture, Faisalabad 38040, Pakistan.

^2^College of Landscape Architecture, Department of Forestry, Sichuan Agricultural University, Chengdu, China.

^3^Plant Production Department, College of Food and Agriculture Sciences, King Saud University, P.O. Box 2460, Riyadh 11451, Saudi Arabia.

^4^Department of Biosystems and Agricultural Engineering (BAE), College of Agriculture and Natural Resources, Michigan State University, East Lansing, MI, USA.

**^*^**Coresspodning author: Muhammad Imran Khan

E-mail: [khanimran1173@yahoo.com](mailto:khanimran1173@yahoo.com); [khanimran@uaf.edu.pk](mailto:khanimran@uaf.edu.pk)

**Table S1.** Treatment details of this study.

| **Treatments** | **Detail** |
| --- | --- |
| NS+P | Plants grown on normal (uncontaminated) soil |
| CS+P | Plants grown on contaminated soil only |
| CS+P+MC | Plants grown on contaminated soil with a microbial consortium only |
| CS+P+nZVI | Plants grown on contaminated soil with nano zero-valent iron particles only |
| CS+P+MC+ nZVI | Plants grown on contaminated soil with a microbial consortium and nano zero-valent iron particles |
| CS | Contaminated soil only |
| CS+MC | Contaminated soil with a microbial consortium only |
| CS+ nZVI | Contaminated soil with nano zero-valent iron particles only |
| CS+MC+ nZVI | Contaminated soil with a microbial consortium and nano zero-valent iron particles |

NS, normal (uncontaminated) soil; CS, soil contaminated with Cd and PHCs; P, plants of sunflower; MC, microbial consortium; nZVI, nano zero-valent iron particles.

**Table S2.** Physicochemical analysis of normal and contaminated soil used in this study.

| **Parameters** | **Normal Soil** | **Contaminated Soil (Before harvest)** | **Contaminated Soil (After harvest)** |
| --- | --- | --- | --- |
| pH | 7.57 ± 2.09 | 7.61 ± 2.18 | 7.85 ± 2.12 |
| Electrical Conductivity (dSm^-1^) | 2.52 ± 1.8 | 3.59 ± 1.05 | 2.64 ± 1.03 |
| Soil Texture | Sandy Loam (sand 50.6%, silt 34.3% and clay 15.1%) | Sandy Loam (sand 50.6%, silt 34.3% and clay 15.1%) | Sandy Loam (sand 50.6%, silt 34.3% and clay 15.1%) |
| Saturation Percentage (%) | 35.0 ± 0.22 | 34.0 ± 0.29 | - |
| Cation Exchange Capacity (cmolkg^-1^) | 12.2 ± 1.32 | 11.0 ± 1.09 | 12.4 ± 2.50 |
| Soil Organic Matter (%) | 0.75 ± 0.11 | 0.72 ± 0.07 | 0.76 ± 1.13 |
| Total Nitrogen (%) | 0.065 ± 0.003 | 0.064 ± 0.002 | 0.079 ± 0.004 |
| Available Phosphorus (mg kg^-1^) | 2.18 ± 1.06 | 2.17 ± 2.09 | 2.86 ± 2.84 |
| Available Potassium (mg kg^-1^) | 61.0 ± 2.29 | 61.0 ± 2.31 | 48.3 ± 2.64 |
| Cadmium (mg kg^-1^) | Not detected | 30 ± 1.88 | 16.5 ± 1.68 |
| Petroleum Hydrocarbons (mg kg^-1^) | Not detected | 6000 ± 9.10 | 3569.8 ± 9.81 |

**Table S3. Average meteorological data (minimum-maximum) of the experiment time (3 months).**

| **Months** | **Sunshine**  **(hours)** | **Temperature**  **(°C)** | **Rainfall**  **(mm)** | **Humidity**  **(%)** | **Evaporation rate (mL)** | **Wind speed (km/h)** |
| --- | --- | --- | --- | --- | --- | --- |
| **August** | 2.50-7.50 | 27.5-34.5 | 2.80-90.2 | 64.0-93.0 | 0.50-6.50 | 1.02-6.30 |
| **September** | 3.00-10.0 | 27.8-34.8 | 8.70-82.5 | 61.8-84.0 | 2.50-7.90 | 1.20-6.50 |
| **October** | 6.50-8.50 | 26.0-31.5 | 0.00-62.0 | 45.0-73.0 | 2.70-4.23 | 1.00-3.30 |

**Characterization of nZVI particles**

The SEM images of green synthesized nano zero valent iron (nZVI) particles showed a highly agglomerated, porous structure with irregular, non-spherical shapes with a rough structure (Figure S1a). The presence of flaky and fibrous features suggests the incorporation of organic compounds from mulberry leaf extract, which likely act as stabilizing and capping agents. The structure reveals multi-layered folds and granular morphology, which may indicate the deposition of iron nanoparticles on a bio-template, possibly formed by the mulberry leaf extract components. The porous and layered morphology indicates a large surface area, which is favorable for adsorption and remediation processes.

The FTIR spectrum of the green-synthesized nZVI particles revealed the presence of functional groups originating from both the iron core and the phytochemical in the mulberry leaf extract used for synthesis (Figure S1b). A broad spectrum band at 3218.2 cm⁻¹ corresponding to the O-H stretching vibrations, indicating hydroxyl groups typically associated with polyphenols. A distinct peak at 2352.8 cm⁻¹ is attributed to C≡N (nitrile) stretching, suggesting the presence of nitrogen containing biomolecules. The peak at 1695.9 cm⁻¹ corresponds to C=C stretching, while the band at 1647.5 cm⁻¹ corresponds to O–H bending. Additionally, the absorption at 1559.9 cm⁻¹ indicates the asymmetric stretching of carboxyl (-COOH) groups. These functional groups confirm the effective capping and stabilization of nZVI particles by bioactive compounds from the plant extract, which facilitated the reduction of iron salts and helped prevent nanoparticles aggregation by forming a protective organic layer.

The XRD analysis confirmed the crystalline nature and phase composition of the green-synthesized nZVI particles (Figure S1c). A distinct peak 2θ = 44.06° corresponds to the (110) plane of body centered cubic (bcc) Fe⁰, indicated the successful synthesis of zero valent iron. The presence of a peak at 2θ = 35.64° attributed to Fe₂O_3_ (hematite), suggested partial surface oxidation, which is commonly observed due to the air exposure during or after the synthesis. Another peak observed at 2θ = 29.31° was assigned to FeO(OH) (iron oxyhydroxide), indicating the development of additional oxidized iron species on the nanoparticles surface. The coexistence of Fe⁰ along with oxidized forms such as Fe₂O_3_ and FeO(OH) supports the typical core-shell structure of green synthesized nZVI particles, where a reactive Fe⁰ core is encapsulated by a stabilizing oxidized shell.

The analysis by Zeta Sizer through Dynamic Light Scattering (DLS) revealed an average hydrometer size of approximately 56 nm (Figure S1d), confirming the successful formation of nanoparticles with the expected nanoscale range.

**Figure S1.** SEM images of green-synthesized nZVI particles showing their irregular structure and uniform distribution (a), FTIR spectrum of nZVI particles showing functional groups associated with plant extract capping and stabilization (b), XRD pattern of nZVI particles confirming the presence of zero-valent iron peaks (c), and zeta sizer analysis of green-synthesized nZVI particles showing the hydrodynamic diameter and size distribution profile (d).

**(a)**

**(b)**

**(c)**

**(d)**

Distribution (%)

| Particle Diameter (nm) | Size distribution Intensity weighted [%] |
| --- | --- |
| 0.209474 | 0 |
| 0.227147 | 0 |
| 0.24631 | 0 |
| 0.26709 | 0 |
| 0.289624 | 0 |
| 0.314058 | 0 |
| 0.340554 | 0 |
| 0.369285 | 0 |
| 0.40044 | 0 |
| 0.434224 | 0 |
| 0.470857 | 0 |
| 0.510582 | 0 |
| 0.553657 | 0 |
| 0.600367 | 0 |
| 0.651018 | 0 |
| 0.705941 | 0 |
| 0.765499 | 0 |
| 0.830081 | 0 |
| 0.900112 | 0 |
| 0.97605 | 0 |
| 1.0584 | 0 |
| 1.14769 | 0 |
| 1.24451 | 0 |
| 1.34951 | 0 |
| 1.46336 | 0 |
| 1.58682 | 0 |
| 1.72069 | 0 |
| 1.86586 | 0 |
| 2.02328 | 0 |
| 2.19397 | 0 |
| 2.37907 | 0 |
| 2.57978 | 0 |
| 2.79743 | 0 |
| 3.03343 | 0 |
| 3.28935 | 0 |
| 3.56686 | 0 |
| 3.86778 | 0 |
| 4.19409 | 0 |
| 4.54793 | 0 |
| 4.93162 | 0 |
| 5.34768 | 0 |
| 5.79885 | 0 |
| 6.28807 | 0 |
| 6.81857 | 0 |
| 7.39383 | 0 |
| 8.01761 | 0 |
| 8.69403 | 0 |
| 9.42751 | 0 |
| 10.2229 | 0 |
| 11.0853 | 0 |
| 12.0206 | 0 |
| 13.0347 | 0 |
| 14.1344 | 0 |
| 15.3268 | 0 |
| 16.6199 | 0 |
| 18.022 | 0 |
| 19.5425 | 0 |
| 21.1912 | 0 |
| 22.979 | 0 |
| 24.9177 | 0 |
| 27.0199 | 0 |
| 29.2994 | 0 |
| 31.7713 | 2.81971 |
| 34.4517 | 4.12548 |
| 37.3583 | 5.46017 |
| 40.51 | 6.72409 |
| 43.9277 | 7.82791 |
| 47.6337 | 8.69568 |
| 51.6524 | 9.26697 |
| 56.0101 | 9.49809 |
| 60.7354 | 9.36262 |
| 65.8594 | 8.85132 |
| 71.4157 | 7.97144 |
| 77.4408 | 6.74552 |
| 83.9741 | 5.20985 |
| 91.0587 | 3.41244 |
| 98.741 | 1.41078 |
| 107.071 | 0 |
| 116.105 | 0 |
| 125.9 | 0 |
| 136.521 | 0 |
| 148.039 | 0 |
| 160.529 | 0 |
| 174.072 | 0 |
| 188.758 | 0 |
| 204.682 | 0 |
| 221.95 | 0 |
| 240.676 | 0 |
| 260.98 | 0 |
| 282.998 | 0 |
| 306.874 | 0 |
| 332.763 | 0 |
| 360.837 | 0 |
| 391.28 | 0 |
| 424.29 | 0 |
| 460.086 | 0 |
| 498.902 | 0 |
| 540.992 | 0 |
| 586.633 | 0 |
| 636.125 | 0 |
| 689.792 | 0 |
| 747.987 | 0 |

**Figure S2.** Pearson correlation matrix among growth, physiological, and biochemical attributes of sunflower plants grown in soil co-contaminated with PHCs and Cd. SG, Seed germination; NOF, Number of flowers; NOSh, Number of shoots; NOS, Number of sepals; NOP, Number of petals; NOL, Number of leaves; RL, Root length; SL, Shoot length; RFW, Root fresh weight; RDW, Root dry weight; SFW, Shoot fresh weight; SDW, Shoot dry weight; TB, Total biomass; LA, Leaf area; CB, Cob diameter; FW, Flower weight; FDW, Flower dry weight; SP, SPAD value; FY, Fluorescence yield; PAR, Photosynthetically active radiation; QY, Quantum yield; ETR, Electron transport rate; MSI, Membrane stability index; RWC, Relative water content; Ca, Chlorophyll a; Cb, Chlorophyll b; Carotenoids; NSh, Nitrogen in shoots; NR, Nitrogen in roots; NSO, Nitrogen in soil; PSh, Phosphorus in shoots; PR, Phosphorus in roots; PSO, Phosphorus in soil; KSh, Potassium in shoots; KR, Potassium in roots; KSO, Potassium in soil; CdSh, Cadmium in shoots; CdR, Cadmium in roots; CdSO, Cadmium in soil; PHCS, Petroleum hydrocarbons in soil. Stars represent significant levels (P ≤ 0.05). Blue and red colors indicate positive and negative correlations, respectively.

(A)

(B)

**Figure S3.** Principal component analysis (PCA) biplot showing the distribution of treatment combination, color indicates the quality of representation (cos2) (A), and contribution of different attributes (B), while in the attributes plot, color reflects the contribution of each attribute in PCA. Dim 1 and Dim 2 explain 81.7% and 18.1% of the total variation, respectively. NS, Normal (uncontaminated) soil; CS, Contaminated soil; P, Sunflower plants; MC, Microbial consortium; nZVI, Nano zero-valent iron particles; SG, Seed germination; NOF, Number of flowers; NOSh, Number of shoots; NOS, Number of sepals; NOP, Number of petals; NOL, Number of leaves; RL, Root length; SL, Shoot length; RFW, Root fresh weight; RDW, Root dry weight; SFW, Shoot fresh weight; SDW, Shoot dry weight; TB, Total biomass; LA, Leaf area; CB, Cob diameter; FW, Flower weight; FDW, Flower dry weight; SP, SPAD value; FY, Fluorescence yield; PAR, Photosynthetically active radiation; QY, Quantum yield; ETR, Electron transport rate; MSI, Membrane stability index; RWC, Relative water content; Ca, Chlorophyll a; Cb, Chlorophyll b; Carotenoids; NSh, Nitrogen in shoots; NR, Nitrogen in roots; NSO, Nitrogen in soil; PSh, Phosphorus in shoots; PR, Phosphorus in roots; PSO, Phosphorus in soil; KSh, Potassium in shoots; KR, Potassium in roots; KSO, Potassium in soil; CdSh, Cadmium in shoots; CdR, Cadmium in roots; CdSO, Cadmium in soil; PCHS, Petroleum hydrocarbons in soil.
